# Weaning-induced alterations in cardiac function: invasive and echocardiographic assessment

**DOI:** 10.1186/cc9591

**Published:** 2011-03-11

**Authors:** A Abdelbary, W Ayoub, Y Nassar, K Hussein

**Affiliations:** 1 Faculty of Medicine, Cairo University, Cairo, Egypt

## Introduction

The aim was to study LV dysfunction during weaning from mechanical ventilation (MV).

## Methods

Thirty invasively MV patients fulfilling the criteria of weaning were shifted to SBT (using low PSV (8 cmH_2_O)) for 30 minutes. Two sets of variables were measured at the beginning and end of the SBT: respiratory rate (F), tidal volume (VT), minute ventilation (VE), peak inspiratory pressure (PIP), PaO_2_/FIO_2 _ratio (P/F ratio); and one reading at the start of the SBT of: airway resistance (Raw), static respiratory compliance (Ceff), maximum negative inspiratory pressure (NIP), (F/VT), arterial blood gases. Weaning failure was defined as: failed SBT, reintubation and/or reventilation or death within 48 hours. Swan-Ganz catheterization was used to obtain the right atrial (RAP), pulmonary artery (PAP), pulmonary artery occlusion (PAOP) pressures, and cardiac index (CI). Echocardiography was used to obtain the LV internal diameter at end diastole (LVIDd) and end systole (LVIDs), ejection fraction (LVEF), E/A ratio, deceleration time (DT) (ms), isovolumetric relaxation time (IVRT), Doppler tissue imaging (DTI) and E/E'.

## Results

Mean age 56.6 ± 15.9 years, 53% were male. Weaning was successful in 76.6% of patients. There was reduction in VT with increase in F and VE (0.53 ± 0.06 vs. 0.45 ± 0.1 l, *P *= 0.0003; 12.5 ± 2 vs. 20.3 ± 7.5, *P *< 0.0001; 6.6 ± 1.5 vs. 8.8 ± 2.4 l, *P *< 0.0001), respectively. P/F_1 was higher than P/F_2 (278 ± 86 vs. 252 ± 74, *P *= 0.005). ABG showed a reduction in PaO_2 _(126 ± 32 vs. 115 ± 29, *P *= 0.01) without change in PaCO_2 _(37.6 ± 6.4 vs. 36.5 ± 6.2, *P *= 0.24). There was a rise in PAOP with insignificant change in RAP, PAP, and CI (12.6 ± 4.7 vs. 14.2 ± 4.7, *P *= 0.003; 6.6 ± 2 vs. 7.2 ± 3, *P *= 0.16; 29.7 ± 7.2 vs. 29.7 ± 7, *P *= 1; 3.2 ± 0.6 vs. 3.22 ± 0.5, *P *= 0.4), respectively. There was a reduction in LVEF with insignificant LVIDd and LVIDs change (66.4 ± 8.1 vs. 64.5 ± 8.4%, *P *= 0.01; 4.83 ± 0.68 vs. 4.7 ± 0.7 cm, *P *= 0.5; 3.1 ± 0.7 vs. 3.12 ± 0.6 cm, *P *= 0.8), respectively. There was no differences between E/A, IVRT, and DT or E/E' at both ends of the trial (1.02 ± 0.38 vs. 1.04 ± 0.37, *P *= 0.6; 95.5 ± 24 vs. 95.8 ± 22, *P *= 0.8; 194.6 ± 30 vs. 195 ± 28 ms, *P *= 0.8; and 9.7 ± 3.1 vs. 10.3 ± 3.5, *P *= 0.09), respectively. E/E' and RAP correlated significantly before and after SBT (*r *= 0.54, *P *= 0.002; and *r *= 0.79, *P *< 0.0001), respectively. Despite insignificant correlation between E/E' and PAOP at the beginning of SBT, there was significant correlation between them at the end of SBT (*r *= 0.6, *P *= 0.001).

## Conclusions

LV dysfunction during weaning is mainly diastolic. Changes in E/E' and RAP and/or PAOP may be the most convenient methods for monitoring diastolic function during weaning from MV.

